# Nematicidal Activity of *Holigarna caustica* (Dennst.) Oken Fruit Is Due to Linoleic Acid

**DOI:** 10.3390/biom10071043

**Published:** 2020-07-14

**Authors:** Sujogya Kumar Panda, Raju Das, Anh Hung Mai, Wim M. De Borggraeve, Walter Luyten

**Affiliations:** 1 Department of Zoology, North Orissa University, Baripada 757003, India; dasraju73@gmail.com (R.D.); walter.luyten@kuleuven.be (W.L.); 2 Department of Biology, KU Leuven, 3000 Leuven, Belgium; 3 Nature’s Foster, P. Box. 41, Shastri Road, Bongaigaon, Assam 783380, India; 4 Department of Chemistry, KU Leuven, Celestijnenlaan 200F, B-3001 Heverlee, Belgium; mai@polymerexpert.fr (A.H.M.); wim.deborggraeve@kuleuven.be (W.M.D.B.)

**Keywords:** *Holigarna caustica*, anthelmintic, Caenorhabditis elegans, linoleic acid, nematicidal, cytotoxicity

## Abstract

*Holigarna caustica* (Dennst.) Oken is used by the tribes of Northeast India for the treatment of intestinal problems. Therefore, the present study was undertaken to investigate the active principles of this plant responsible for its anthelmintic activity, using bioassay-guided fractionation. An ethanol extract of *H. caustica* fruit was fractionated on a silica gel column, followed by HPLC, while nematicidal activity was followed throughout on *Caenorhabditis (C.) elegans* as a model organism. Our study constitutes the first nematicidal report for this plant. Bioassay-guided purification led to the isolation of one compound (IC_50_ = 0.4 µM) as the only active constituent in the most active fraction. The compound was identified as linoleic acid based on spectroscopic data (^1^H and ^13^C NMR and ESI-MS). No cytotoxicity was observed in the crude extract or in linoleic acid (up to 356 µM). The results support the use of *H. caustica* for the treatment of intestinal problems by traditional healers in India.

## 1. Introduction

Neglected tropical diseases (NTDs) are a group of 20 diseases considered as the most devastating infections in developing countries (https://www.who.int/neglected_diseases/diseases/en/). They comprise both chronic parasitic and bacterial infections, but the most common causative agents of NTDs are parasitic helminths [1]. A recent survey on parasitic worms (soil-transmitted helminths) of the gastrointestinal tract estimated over 1.5 billion people to be infected, attesting to its major global importance (https://www.who.int/news-room/fact-sheets/detail/soil-transmitted-helminth-infections). It is also a serious problem in livestock production, causing significant economic losses and threatening food security [2,3]. Nematodes are the most diverse taxon, with an estimated 100,000 to 1,000,000 species (https://www.csiro.au/en/Research/Collections/ANIC/Insect-research/Roundworms-Research). Intestinal parasitic nematodes mainly include: *Ascaris lumbricoides*, *Trichuris trichiura*, *Ancylostoma duodenale*, *Necator americanus*, *Strongyloides stercoralis*, *Enterobius vermicularis*, and *Capillaria philippinensis* as the most common in humans, infecting approximately 2 billion people worldwide [4]. Control strategies of helminths rely mostly on a limited number of synthetic anthelmintic drugs, whereas no vaccine is available for humans. Only four main anthelmintic drugs are used for treating human intestinal nematodes: pyrantel pamoate, albendazole, mebendazole, and levamisole. The progress of anthelmintic drug discovery is very slow [4]. The anthelmintic drug tribendimidine has entered human clinical trials (in China in 2007) [5], followed more recently by “emodepside” in the DRC and Ghana (https://www.dndi.org/diseases-projects/portfolio/emodepside/). To the best of our knowledge, no novel drug candidates against human intestinal nematodes are in clinical development at present.

Resistance to anthelmintic drugs is on the rise, in part due to their injudicious use. Although inexpensive by Western standards, they may be difficult to afford by small-scale farmers in developing countries. They tend to rely on local plants, whose efficacy is typically poorly supported by scientific evidence. Further, little is known about the optimum dose and treatment frequency with such plants, as well as potential toxicities for the treated animal or those who consume its products. Therefore, alternative control strategies are urgently needed.

Plant-based medicines have been an integral part of the traditional health care system in most parts of the world for thousands of years [6]. The global demand for phytomedicine is rapidly increasing, and more work is needed to discover potent antiparasitic drug(s) based on natural products [7]. Worldwide, several medicinal plants have been reported as anthelmintic or antiparasitic [4,7,8]. In the past 15 years, over “three dozen anthelmintic compounds were isolated from medicinal plants, most of which are used traditionally to treat gastrointestinal nematodes” [4]. India has a rich tradition of herbal medicine for the treatment of various infectious diseases. For millennia, traditional healers have used plants to combat parasitic disease. Ethnomedicine is still widely practiced in India, and traditional healers have a sound knowledge of infections caused by various intestinal worms, their mode of transmission, and typical symptoms viz. abdominal pain and growth retardation [9]. Therefore, selecting plants based on healers’ knowledge is an attractive approach to identify relevant bioactive compounds, and such studies also help the scientific validation of these traditional practices, which has mostly been lacking.

*Holigarna (H.) caustica* (Dennst.) Oken belongs to the Anacardiaceae family, and has not been investigated for anthelmintic properties. A recent study by Adnan and coworkers [6] documented the use of this plant by indigenous communities of the Chittagong Hill Tracts of Bangladesh for the treatment of a variety of painful conditions such as eye irritation, inflammation, arthritis, skin diseases, cuts, and wounds [10]. Recently, the plant was found to be effective to control multidrug-resistant *S. aureus*, including in biofilms [11]. A previous study documented that the tribes in Northeast India use 37 plant species belonging to 26 families, where the most common medicinal uses are skin infections and fever (including malaria) (16 plants), followed by worm infections (10 plants) [12]. Amongst these ten plants, *H. caustica* is one of the plants for the treatment of intestinal problems (as cold or hot infusion). Other traditional uses reported include tumors, piles, and skin diseases [12]. Therefore, the aim of this study is to test this plant for anthelmintic activity, and find the active principles responsible for this activity using bioassay-guided fractionation.

## 2. Materials and Methods

### 2.1. Chemicals and Reagents

Acetone and n–hexane, both of analytical grade, were purchased from Sigma–Aldrich Co. (USA). Absolute ethanol was purchased from Fisher Chemicals (Loughborough, UK). Sterile deionized water was produced by Milli-Q water purification system (Millipore, Brussels, Belgium). Ammonium chloride, calcium chloride, cholesterol, dextrose, dimethyl sulfoxide (DMSO, molecular biology grade), levamisole hydrochloride, linoleic acid, magnesium sulphate, potassium dihydrogen phosphate, di–sodium hydrogen phosphate, sodium chloride, sodium hydroxide, and sucrose were all purchased from Sigma–Aldrich (St. Louis, MO, USA). Stock solutions of linoleic acid were prepared in DMSO and stored in aliquots at 4 °C till use.

### 2.2. Plant Collection and Extraction

The plant *H. caustica* was collected from the Chirang Reserve Forest (CRF), Northeast India, and identified by a taxonomic expert at North Orissa University, Baripada, where a voucher specimen (RDS-1132) was deposited. Fruits were dried in an oven at 50 °C, while leaves were separately dried at ambient temperature to maintain their green color and volatile oils, if present [13]. The dried raw botanical material was ground to a fine powder. Small-scale extraction was performed using four solvents: hexane, ethanol, acetone, and water, as described earlier. After extractions, 1 mL aliquots of the crude extracts were transferred into pre-weighed 2 mL Eppendorf tubes, then dried in a Savant™ SpeedVac™ apparatus, weighed, and stored in a cold room at 4 °C. Just before a biological assay, the dried residue was dissolved in DMSO (for organic extracts) or water (for the aqueous extract): the final concentration in the assay was 1000 µg/mL. For more details, see Panda et al. [14]. Ethanol was selected for a large-scale extraction. The dried fruits of *H. caustica* (100 g) were ground to a fine powder and mixed with 500 L of ethanol in a screw-capped glass bottle, shaken, and sonicated in a sonication water bath for 4 times 30 min over 24 h. The decoction was decanted through filter paper (185 mm, Macherey-Nagel GmbH & Co. KG, Germany), and the process was repeated 3 times to maximize extraction. The material was concentrated under reduced pressure using a Büchi rotavapor R-100.

### 2.3. Bioassay-Guided Fractionation and Isolation

The dry residue of the ethanol extract (14 g) was chromatographed on silica gel 60 (400 g, 37 cm × 5 cm glass column). The column was initially eluted with hexane (500 mL), followed by ethyl acetate/hexane mixtures of increasing polarity (ethyl acetate/hexane 1:3, 1:1, 3:1, 1:0, all *v*:*v*). The polarity of the eluting solvent was sequentially increased further by using methanol/ethyl acetate (0.5:9.5, 1:9, 1.5:8.5, 2:8, 4:6, 6:4, 8:2, 1:0) and acetic acid/methanol (0.5:9.5, 1:9). Fourteen fractions of 500 mL each were collected, concentrated, and tested for bioactivity.

### 2.4. HPLC-DAD Analysis

HPLC was performed on a Shimadzu, LC-20AT system (model DGU 20A3) as described earlier [15]. Briefly, 2 mL of sample was injected and separated at a flow rate of 4 mL/min on a reverse-phase HPLC column: Sunfire^TM^ prep C18 (10 mm × 250 mm, 5 µm, Waters). The mobile phase was composed of solvent (A): H_2_O with 0.1% trifluoroacetic acid (0–20%), and solvent (B): acetonitrile (LC-MS *Chromasolv*^®^, Fluka) with 0.1% trifluoro acetic acid (80–100%). Eluate fractions were collected every minute, dried, dissolved in DMSO, and tested for nematicidal activity. The peaks in the chromatogram corresponding to the active fractions were then collected manually, dried, and their nematicidal activity was verified. Then, the most active peak was collected for mass spectrometry (MS) and nuclear magnetic resonance (NMR) analysis.

### 2.5. Mass Spectrometry (MS)

The analysis was performed on a Q Exactive orbitrap mass spectrometer (Thermo Scientific, San Jose, CA) as described earlier [16]. From the high-accuracy masses the best-matched chemical formulas were inferred with ChemCalc [17]. The proposed chemical formulas were then used to search the Universal Natural Products Database (http://bioinf-applied.charite.de/supernatural_new/index.php?site=home).

### 2.6. NMR Spectroscopy

^1^H and ^13^C NMR spectra were recorded on an FT-NMR spectrometer (Bruker, Fallanden, Switzerland) Avance II operating at 600 MHz. All experiments were performed in deuterochloroform solution and at 288 K; chemical shifts are expressed in *δ* scale (ppm) using tetramethylsilane as an internal standard and coupling constants *J* are in hertz (Hz).

### 2.7. Nematicidal Activity

The assay was carried out in a 96-well microplate (flat-bottom, TPP Techno Plastic Products AG, Switzerland). Fresh L4 stage larvae were collected in M9 buffer and adjusted to approximately 5000 larvae/mL. The wells were filled with 190 µL of overnight cultured *E. coli* OP50 (O.D. 600 = 0.5) and subsequently 10 µL of worm suspension was added to get ~45 L4 larvae per well. Subsequently, 1 μL of test sample (stock solution or dilution thereof in DMSO at different concentrations) was added to each well; DMSO (1 μL) was used in a separate well as a solvent control. The movement of worms in each well of the 96-well plate was recorded for 20 h at 20 °C using a WMicroTracker (Phylumtech, Argentina) apparatus. The percentage of the average movement over 20 h in the presence of test samples with the extract, compared with the DMSO control (0.5%), was used to estimate the relative nematicidal activity. Levamisole (final concentration 50 µM) was systematically used as a positive control. After 20 h of incubation, all wells were observed under a microscope to distinguish cidal from non-cidal activity. For more details on the culture, maintenance, and synchronization of *C. elegans*, see Panda et al. [14].

### 2.8. Cytotoxicity Test

Electrical impedance measurements were performed on a proprietary electrical impedance spectroscopy device (CellSine, http://cellsine.com/) using 96-well electronic microtiter plate. The BJ-5ta (ATCC CRL-4001) cell line was routinely grown in a humidified 5% CO_2_ incubator in Dulbecco’s modified Eagle’s medium with 10% fetal bovine serum (FBS) and 1% penicillin/streptomycin (P/S). For time-dependent cell response profiling, 50 µL of culture medium was added to each well to obtain background readings. Next, 50 µL of a cell suspension was transferred to the 96-well electronic microtiter plate (20,000 cells/well), followed by 24 h incubation at 37 °C in a humidified atmosphere at 5% CO_2_ (Sanyo, MCO 17 AIC, Japan). After 24 h, the culture medium was replaced by the medium without FBS, followed by 3 h incubation. The medium was again replaced by fresh medium to which 10 µL of test solution was added (final DMSO concentration 0.5%). Cells were monitored with a temporal resolution of 8 s per well. At the start of the experiment, each well was measured once before a test solution was added. Raw impedance data were converted into a dimensionless parameter called cell index (CI), which reflects the relative change in measured electrical impedance to represent cell status [18].

### 2.9. Statistical Analysis

All assays were carried out at least in duplicate and repeated at least once for confirmation. Data from dose–response experiments are represented as the percentage of inhibition compared with the solvent control, and analyzed with Prism^TM^ (GraphPad Prism 5.0 Software Inc., San Diego, CA, United States). The IC_50_ for each growth condition was calculated by fitting the data to a non-linear least-squares sigmoid regression curve, fixing the top and bottom of the curve at 100 and 0 percent, respectively. The IC_50_ corresponds to the concentration that would yield an inhibition of 50% [19].

## 3. Results and Discussion

### 3.1. Fruit Extracts of H. caustica Prevent the Movement of C. elegans

Four solvent extracts were tested against *C. elegans,* and all three organic ones (hexane, acetone, and ethanol) were found active (>50% inhibition). Although the hexane extract was more potent, its yield was much lower compared with ethanol; therefore, we prepared a large-scale ethanol extract of *H. caustica* and subjected the dried residue to bioassay-guided fractionation.

*Holigarna* Buch.-Ham. ex Roxb., is a genus of large trees, confined to tropical evergreen forests of the Indo-Malayan region, ranging from its western coast eastwards through the Andaman Islands into Myanmar. Only seven species are reported worldwide (http://www.theplantlist.org/tpl1.1/search?q=holigarna), and they are not well studied for medicinal use or phytochemistry. *Holigarna caustica* is critically endangered at Baraiyadhala National Park, Bangladesh [20]. Recently, Adnan et al. [10] documented 40 compounds from this plant using GC-MS. Among the 40 compounds, more than a dozen have previously documented bioactivity (analgesic and anti-inflammatory properties) such as α-tocopherol, *n*-hexadecanoic acid, oleate, 9,12-octadecadienoic acid (*Z,Z*), 9,12-octadecadienoic acid (*Z,Z*), 9-octadecenoic acid (*E*), methyl ester, neophytadiene, phytol, stigmast-5-en-3-ol, stigmasterol, squalene, γ-tocopherol, and γ-sitosterol. Jadav et al. [21] isolated 44 chemical constituents from the stem bark, leaves, latex, and fruits of *H. grahamii* using GC-MS. The most abundant components were 1,2,3-benzenetriol, butanoic acid, furanmethanol, 2- furancarboxaldehyde, quinic acid, and 2-ethylhexyl ester [21], but their bioactivity was not studied.

### 3.2. Bioassay-Guided Purification of Nematicidal Compounds

**Figure 1:**
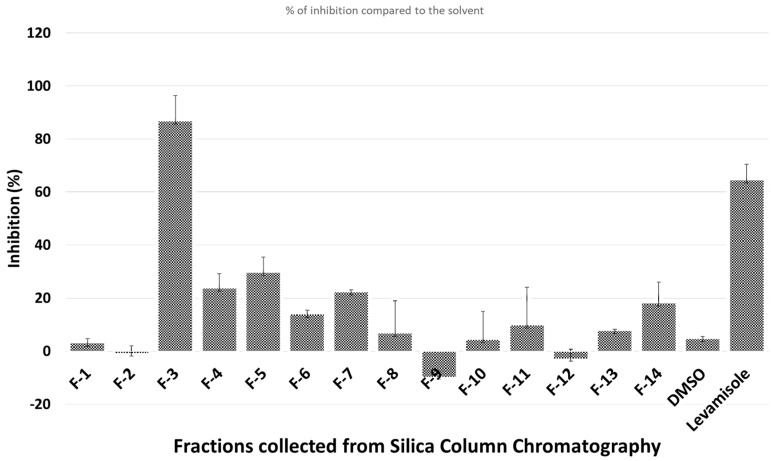
Nematicidal effects (activity based on movement of *Caenorhabditis (C.) elegans* detected by infrared microbeams) of different fractions collected from silica gel column.

The crude ethanol extract of *H. caustica* fruit (extraction yield = 14%) was first separated on a silica gel column; the nematicidal activity against *C. elegans* of different fractions is shown in Figure 1.

**Figure 2:**
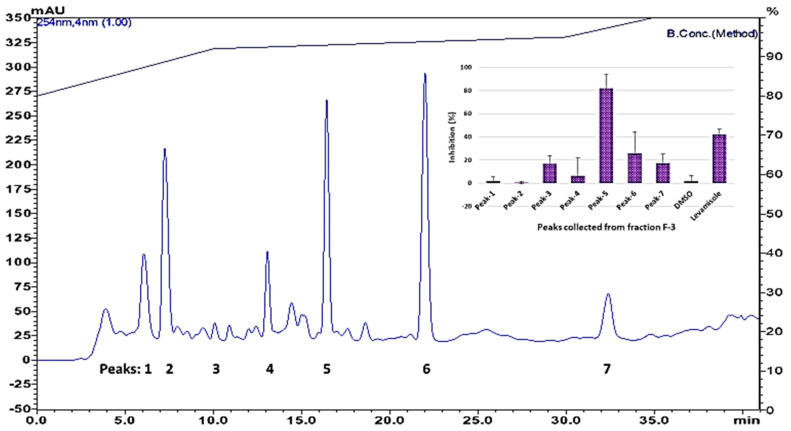
HPLC chromatogram of silica column fraction 3, and nematicidal activity of chromatographic peaks. Top right corner, data presented as nematicidal effects (activity based on movement of *C. elegans* detected by infrared microbeams) of individual peaks collected from HPLC.

Only fraction 3 (F-3), which elutes with ethyl acetate/hexane, 50:50, had a clear nematicidal effect. Thus, F-3 was further separated on HPLC to isolate the active compounds. Only one chromatographic peak (Peak-5) showed significant activity (Figure 2).

**Figure 3:**
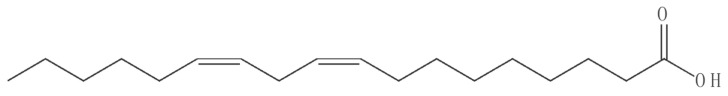
Structure of bioactive compound isolated from *Holigarna (H.) caustica* (linoleic acid).

Peak-5 from fraction 3 (eluting at 16 min) was analyzed by MS; the proposed molecular formula (C_18_H_32_O_2_) is based on negative-ion *m*/*z* 279.2322 [M-H]^−^ (calcd. 279.2322 for C_18_H_32_O_2_). The tentative identification by MS was confirmed by NMR as linoleic acid (structure is shown in Figure 3) by comparison of its spectroscopic data (^1^H and ^13^C NMR) with those reported in the Biological Magnetic Resonance Data Bank. ^1^H NMR (600 MHz, CDCl_3_, 288 K): *δ* 5.41–5.31 (m, 4H), 2.77 (t, *J* = 6.8 Hz, 2H), 2.35 (t, *J* = 7.6 Hz, 2H), 2.05 (q, *J* = 7.0 Hz, 4H), 1.66–1.61 (m, 2H), 1.36–1.25 (m, 14H), 0.89 (t, *J* = 6.8 Hz, 3H). ^13^C NMR (150 MHz, CDCl_3_, 288 K): *δ* 179.77, 130.21, 130.02, 128.00, 127.84, 33.96, 31.51, 29.57, 29.34, 29.15, 29.07, 29.00, 27.18, 27.17, 25.58, 24.63, 22.58, 14.12.

Our study demonstrates that linoleic acid is the major compound responsible for the nematicidal properties from the ethanolic extracts of *H. caustica* fruit. Although extracts with other solvents (acetone and hexane) were also active, we believe that the same compound may well be responsible, as linoleic acid is highly soluble in acetone, benzene, diethyl ether, and ethanol (https://en.wikipedia.org/wiki/Linoleic_acid). Conversely, the water extract was not found to be active, probably because the solubility of linoleic acid in water is too low (0.139 mg/L). We found some other fractions of the silica gel - and C18 column that showed weak activity but did not pursue those. They may contain linoleic acid or other nematicidal compounds present in much lower amounts. We cannot exclude that such minor compounds could synergize amongst themselves or with linoleic acid. This is the first demonstration of the nematicidal activity of *H. caustica* extracts. Our study suggests that fruits of this tree may be a good choice for developing an anthelmintic botanical preparation, as the fruits are not used for any other purpose.

### 3.3. Nematicidal Activity of Linoleic Acid

**Figure 4:**
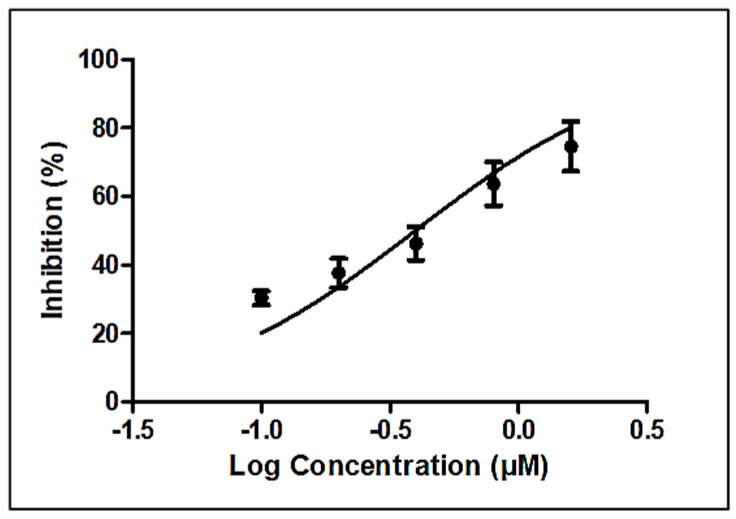
Estimation of nematicidal IC_50_ of linoleic acid (Sigma, St. Louis, MO, USA).

Linoleic acid (>99%) was purchased from Sigma, USA (L2376), and studied for its nematicidal and cytotoxic activities. Dose–response curves yielded an IC_50_ of 0.4 µM (95% confidence interval, 0.30 to 0.52) or approximately 0.2 µg/mL (Figure 4), which compares favorably with that of the established anthelmintic levamisole (6.4 ± 0.3 µM) [22].

*C. elegans* is an attractive animal model for studies of nematicidal properties. This model organism is easy and inexpensive to grow, and its well-studied developmental biology, simple anatomy, short lifespan, well-annotated genome, and ease of genetic analysis allow for studies of diverse biological processes, including those related to human nutrition and disease [23].

The anthelmintic effect of linoleic acid has been demonstrated earlier. Linoleic acid extracted from the stem and root of *Mucuna aterrima* demonstrated nematicidal activity in *Meloidogyne incognita* J2s, and inhibited egg hatching [24]. Linoleic acid is one of the constituents of *Clonostachys candelabrum* active against *Haemonchus contortus* [25], and it was the only detectable nematicidal agent in the mycelial extracts of several predacious fungi of the genus *Arthrobotrys* [26]. Linoleic acid was one of several nematicidal fatty acids isolated from cultures of two Basidiomycetes (*Pleurotus pulmonarius* and *Hericium coralloides*) in a screen using *C. elegans*: the LD_50_ was estimated between 5 and 10 ppm, which corresponds to 5–10 µg/mL [27]. The estimated IC_50_ in our hands is much lower, i.e., ~0.2 µg/mL, but the reason for this discrepancy is not clear. Another study suggests that a plant fraction containing mostly medium-chain fatty acids and phenolic acids has anthelmintic activity on *C. elegans* and *Haemonchus contortus* [28]. A structure–activity analysis of the nematicidal activity of fatty acids against the cyst nematode *Heterodera zeae* showed that the activity depends on several factors, such as chain length, and number as well as position of double bonds [29].

### 3.4. Cytotoxicity of Linoleic Acid

**Figure 5:**
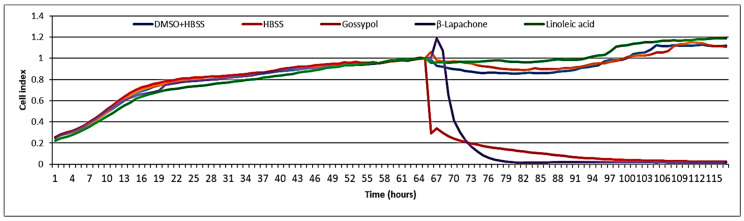
Cytotoxicity test of linoleic acid on BJ5ta cells.

No cytotoxicity was observed when tested at 100 µg/mL (i.e., 356 µM) on the BJ5ta cell line using electrical impedance spectroscopy (Figure 5). The well-known cytotoxic compounds gossypol and β-lapachone were used as positive controls. Melariri et al. [30] investigated the antiplasmodial activity of linoleic acid against *P*. *falciparum*. They found that several fatty acids, including linoleic acid, were not cytotoxic to Chinese hamster ovary (CHO) cells, but were good inhibitors (IC_50_ = 6.8 μg/mL) of *P. falciparum* [30]. In the present study, the cytotoxicity was measured in BJ-5ta cells (normal fibroblast immortalized with hTERT), but even at the highest concentration tested (100 µg/mL), linoleic acid did not show any cytotoxic effects. During an antiviral activity study, the same extract was also tested for cytotoxicity on rhabdosarcoma cells, and no cytotoxicity was observed when tested at the highest concentration 100 µg/mL [12]. Several studies report on the in vitro or in vivo toxicity of linoleic acid in various models, including humans. Shultz et al. [31] and Cunningham et al. [32] found linoleic acid not to be cytotoxic to normal human and MCF-7 breast cancer cells. However, a few authors also reported that chronic high intake of linoleic acid substantially raises the risks of breast, colorectal, or prostate cancer in humans [33,34]. Nematode treatment, however, would typically be brief, so that chronic toxicity is not a major concern.

### 3.5. Suitability of H. caustica Fruit or Linoleic Acid as Anthelmintic

Linoleic acid is an essential fatty acid for humans, and is obtained from the diet. The total concentration of linoleic acid in human plasma is around 2 mM [35,36] (around 550 µg/mL), far above the IC_50_ values that we determined. However, most of it is bound to plasma proteins like albumin, so that the free concentration is only around 10 nM (2.8 ng/mL), far below the IC_50_.

It may be possible to reach much higher levels in the gut, so that treatment of intestinal parasites with dietary linoleic acid may be envisaged. Based on our purification, we estimate that the linoleic acid content of *H. caustica* is 660 µg/gram of dry weight of fruit. If the fruit contains ~50% water, then 330 µg/gram of linoleic acid will be present in the fresh fruit. If we assume that 100 g of fresh fruit corresponds to one serving, then one such serving contains 330 µg of linoleic acid. If the fruit was fully digested, and assuming a small intestinal volume of 50–100 mL, then a concentration of 3.3 µg/mL will be reached, which is above the IC_50_ of linoleic acid.

Linoleic acid will readily oxidize in air. Stable oral formulations of linoleic acid have been developed [37,38], for instance, by encapsulation and/or addition of anti-oxidants. Many plant extracts contain antioxidants that may stabilize linoleic acid: *H. caustica*, for instance, has strong anti-oxidant properties [39].

### 3.6. Mechanism of Action of Linoleic Acid

The antiparasitic mechanisms of linoleic acid are not clear. “It has been suggested that the nematicidal activity of fatty acids is due to adverse interference with the nematode cuticle or hypodermis via a detergent (solubilization) effect, or through direct interaction of the fatty acids with the lipophilic regions of target plasma membranes” [40,41]. Linoleic acid can affect ionotropic neurotransmitter receptors, like gamma-aminobutyric acid (GABA) and N-methyl-D-aspartate (NMDA) receptors [42], which are targets of some common anthelmintics. Further studies using a panel of mutant *C. elegans* lines resistant to currently used anthelmintics could be used to establish whether the molecular target of linoleic acid is the same as that of the established anthelmintics. If not, *C. elegans* mutants resistant to linoleic acid could be generated to identify the mechanisms of action, which may provide new drug targets for rational drug discovery efforts. Further, identifying the mechanism of resistance may help to extend the useful lifespan of anthelmintic compounds [43]. For the next generation of anthelmintic drugs, a novel mechanism of action is preferred to circumvent resistance. Additionally, such drugs may act synergistically with current anthelmintics, which may lead to new drug combinations. Only two compounds, tribendimidine and emodepside, have entered human clinical trials in the last 35 years for treating helminth infections. According to the World Health Organization and the Bill and Melinda Gates Foundation, there is a great need for new anthelmintic treatments.

## 4. Conclusions

In conclusion, this study constitutes the first report on the isolation and identification of linoleic acid from *H. caustica* fruit, and the demonstration that it is the major compound responsible for its nematicidal properties. Linoleic acid might be developed as an effective drug to control intestinal parasites as well as for the management of soil nematodes, with little toxicity risk. Further research is required to evaluate the mode of action of this compound. In addition, more experiments are needed to evaluate the effects of linoleic acid and its analogues on common parasites with in vivo studies.

## Chemical Compounds Studied in This Article

Linoleic acid (PubChem CID: 5280450).

## Abbreviations

ATCC	American Type Culture Collection, Manassas, Virginia, USA
BJ5ta	Normal fibroblast immortalized with hTERT
CRF	Chirang Reserve Forest
DMSO	Dimethyl sulfoxide
ESI	Electrospray ionization
FBS	Fetal bovine serum
HPLC	High performance liquid chromatography
IC_50_	50% Inhibitory concentration
MS	Mass spectrometry
NMR	Nuclear magnetic resonance
NTDs	Neglected tropical diseases
TFA	Trifluoro acetic acid
WHO	World Health Organization
